# Accumulation of Microcystin-LR in Grains of Two Rice Varieties (*Oryza sativa L.*) and a Leafy Vegetable, *Ipomoea aquatica*

**DOI:** 10.3390/toxins11080432

**Published:** 2019-07-24

**Authors:** Menuja M Wijewickrama, Pathmalal M Manage

**Affiliations:** Center for Water Quality and Algae Research, Department of Zoology, University of Sri Jayewardenepura, Gangodawila, Nugegoda 10250, Sri Lanka

**Keywords:** microcystins, *Oryza sativa*, *Ipomoea aquatica*, human health risk, tolerable daily intake

## Abstract

The potential transfer of microcystin-LR (MC-LR) to humans via crop plants irrigated with MC-contaminated water is causing serious concern. In this study, two *Oryza sativa* variants, a hybrid (BG358), a traditional (Suwandel) variety, and a leafy green vegetable crop, *Ipomoea aquatica*, were exposed under laboratory conditions to natural blooms of *Microcystis aeruginosa* sampled from a hypereutrophic lake contaminated with MC-LR (3,197.37 ± 1.04 µg/L). Field samples of *O. sativa* and *I. aquatica* were collected from farmlands that had been irrigated from a reservoir, containing MC-LR (180 µg/L). MC-LR was quantified by high performance liquid chromatography followed by photodiode-array detection (HPLC-PDA). From the laboratory study, we calculated the potential human health exposure from BG358, Suwandel and *I. aquatica* as 2.84 ± 0.01, 0.22 ± 0.01, and 0.06 ± 0.01 µg/kg of body weight/day, respectively, whereas the potential health exposures from BG358, Suwandel and *I. aquatica* collected from the field were 0.10 ± 0.01, 0.009 ± 0.005, and 0.03 ± 0.01 µg/kg of body weight/day, respectively. In certain instances, the results exceeded the World Health Organization’s (WHO) tolerable daily intake of MC-LR, posing a potential health risk to humans. Thus, our results emphasize the importance of continuous screening programs for cyanotoxins in edible plants in the future to prevent the consumption of contaminated crops.

## 1. Introduction

The eutrophication of freshwater bodies has led to harmful cyanobacterial blooms that have the potential to release cyanotoxins [1,2]. Cyanotoxins are secondary metabolites categorized chemically into cyclic peptides, alkaloids, or lipopolysaccharides (LPS), and functionally as hepatotoxins, neurotoxins, and dermatotoxins [1,2,3,4,5,6,7,8]. However, their exact role within cyanobacteria and their persistence in the environment is not clearly understood [1,9]. Among different cyanotoxin varieties, microcystin (MC) is the most dominant and the most toxic in the aquatic environment [3,4]. *Anabaena, Fischerella, Gloeotrichia, Nodularia, Nostoc, Oscillatoria,* members of *Microcystis,* and *Planktothrix* are some of the MC-producing genera of cyanobacteria that produce extremely water soluble and non-volatile MCs [1,4,5,6,7]. There are over 100 recorded hepatopeptide MC congeners such as MC-LR, -RR, -YR, and -LW, among which MC-LR is the most common and most toxic [8,9]. Thus, the World Health Organization (WHO) established a provisional guideline limit of 1 μg/L for MC-LR in drinking water and 0.04 μg/kg of body weight/day as the tolerable daily intake (TDI) for humans [9,10]. 

Incidences of MC toxicity and poisoning have been recorded worldwide, and liver damage is considered to be the major human impact of MC-LR [4,6,9,10,11,12,13,14,15,16]. In vitro studies have reported potential damage by MC-LR to other vital organs such as kidney, thymus, male reproductive organs, and intestines [17,18,19,20,21,22,23,24,25]. MC-LR acts as a strong inhibitor of protein phosphatase enzyme 1 and 2A and related enzymes, and these are critical for essential cellular functions in higher plants and animals [26,27]. MCs have the potential to induce lesions in the mitochondria of kidney cells, thus leading to kidney injuries [28,29]. MC-LR is known to be responsible for low-impact health implications in animals such as dermatitis, irritations, asthma-like symptoms, gastroenteritis, and related diseases and symptoms resembling hay fever and allergies [4,9,10,29,30].

The nephrotoxic effects of MC-LR may also be an important factor responsible for a chronic kidney disease of unknown etiology (CKDu) in Sri Lanka [29,31,32,33]. CKDu is mainly prevalent in the North Central Province and to a lesser extent in some parts of Uva, as well as the Eastern and North Western Provinces [34,35]. The MC-LR contamination of dug wells and surface water bodies, which are used to extract water for drinking and irrigation, is significantly correlated with the CKDu patient distribution, adding considerable support to an MC-LR-induced nephrotoxicity explanation for CKDu [32,33,34,35,36,37]. 

MC-LR and other MC variants may accumulate in living organisms in substantial concentrations via the food chain, drinking water, and direct exposure through skin and the respiratory pathway [4,10,11]. The hydrophilic and stable properties of MCs allow them to be bioaccumulated, bioconcentrated, and biomagnified along the food chain, thus increasing the threat to higher trophic levels including humans [4,6,9]. Humans might be exposed to MC-LR via the consumption of crops irrigated with MC-contaminated water. Cyanotoxin accumulation in edible storage organs, such as stems, leaves, fruits, seeds, and corms of agricultural crops via direct irrigation and spray irrigation has been recorded [38,39,40,41,42].

Though almost all reservoirs in Sri Lanka are used for agricultural purposes, intensive catchment modifications by human activities have accelerated the eutrophication of irrigation reservoirs, thus leading to toxic cyanobacterial blooms [36,37,43,44]. Thus, the main cyanotoxin found in irrigation reservoirs in Sri Lanka is MC-LR, so consumption of crop varieties grown in MC-LR-contaminated environments could potentially lead to serious health issues [31,43,44]. The Padaviya reservoir in North Central Province, Sri Lanka is one such eutrophic reservoir that is the major water source for paddy rice cultivators and other farmlands in Padaviya [32,36,37,45]. An additional cause for concern is that the Padaviya region has recorded the most CKDu cases in the country [32,33,34,35]. The MC-LR-producing cyanobacteria *Microcystis aeruginosa* is the dominant cyanobacteria in the reservoir, with an MC-LR concentration of 55–65 μg/L during the non-blooming season [31,36,44]. The concentration of MC-LR increases to approximately 180 μg/L (pers. obs.) during the dry season when *M. aeruginosa* blooms occur, and this covers the entire rice growing season (from May to August) [18,31,34]. 

Rice (*Oryza sativa*) is a staple food source and is the most cultivated terrestrial crop in Sri Lanka [45,46]. Besides rice, most Sri Lankans also consume *Ipomoea aquatica*, commonly known as ‘Kangkong,’ which is a green, leafy vegetable. It is among the 10 main leafy vegetables consumed in South Asia, with an average annual per capita consumption of 2.2–3.6 kg/y [47]. Both *I. aquatica* and *O. sativ*a require high amounts of water, which is mainly provided by irrigation reservoirs. Cyanobacterial blooms in irrigation reservoirs occur in the dry season, which covers the entire rice cultivation season, so MC-LR can potentially accumulate in rice grains. Roots of rice also have a symbiotic relationship with some of the MC-LR-producing cyanobacteria, such as *Anabaena, Nostoc,* and *Ossciallatoria,* which is an alternate route of MC-LR entrance into rice grains [48,49,50]. Frighteningly, CKDu predominates in the major agricultural areas in Sri Lanka [32,33,34,35].

Therefore, the objectives of the present study were to investigate the accumulation of MC-LR in two major agricultural crops—rice (*O. sativa*) and a leafy green vegetable (*I. aquatica*)—in Sri Lanka, irrigated directly from an MC-LR-contaminated agricultural reservoir, and to determine the accumulation potential of MC-LR in crops when irrigated with fresh bloom-contaminated water. The risk to human health via consuming MC-LR contaminated crops was also evaluated.

## 2. Results

**Figure 1:**
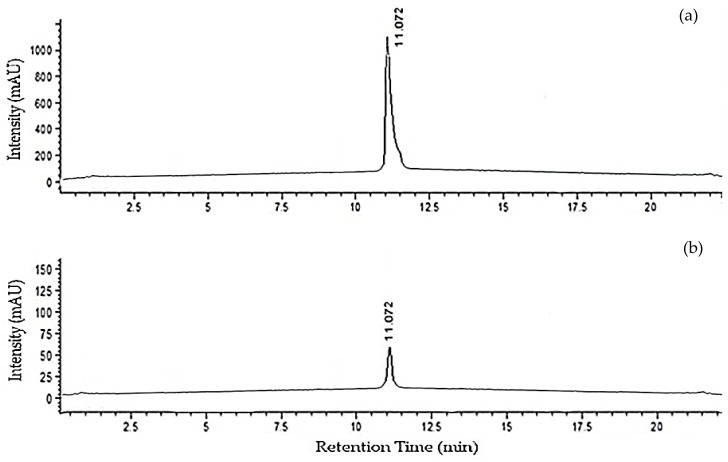
Representative HPLC chromatograms of microcystin-LR (MC-LR) obtained from (**a**) a 100 ppm standard solution; (**b**) a field sample of the *Oryza sativa* variant, BG358 with MC-LR concentration of 20.97 ± 0.31 µg/kg.

MC-LR accumulation levels in *O. sativa*, variants BG358 and Suwandel, and *I. aquatica* were measured using HPLC from samples exposed in both the laboratory and field studies. Typical MC-LR chromoatograms of the standard solution and in a *O. sativa*, BG358 sample from the field study are depicted in Figure 1.

### 2.1. Laboratory Studies

Plants were irrigated daily with fresh water containing *M. aeruginosa* with MC-LR concentrations of 3,197.37 ± 1.04 µg/L. This contaminated water was obtained from 10 cm below the surface of a hypereutrophic lake, Beira Lake, Sri Lanka. Negative controls of three plant types were irrigated with chlorine-free tap water. All plants treated with cyanobacteria-contaminated water contained very high levels of MC-LR (~450 µg/kg) after four months of treatment. BG358 had the highest accumulated MC-LR concentration in the laboratory study (Table 1). All the plant types tested showed a significant difference in the MC-LR accumulation in their edible tissues even though they were exposed to same MC-LR concentrations (ANOVA, *p* < 0.05). 

Based on the average annual consumption figures for these three crops, these values correspond to a potential human exposure of 2.84, 0.22 and 0.06 µg/kg of body weight/ day MC-LR for *O. sativa*, BG358, Suwandel, and *I. aquatica*, respectively. (Table 1) [47,51,52]. 

### 2.2. Field Studies

All plants were exposed to MC-LR via irrigation from the Padaviya reservoir (180.17 ± 0.59 µg/L). The levels of MC-LR in edible tissue in each crop type were considerably lower than in the laboratory samples, ranging from 18.19 to 132.86 µg/kg (ANOVA, *p* < 0.05). Though both varieties of rice were exposed to the same concentration of MC-LR, the accumulation of MC-LR was higher in the grains of the hybrid rice variety BG358 (Table 1). Interestingly, while *I. aquatica* had the lowest tissue concentration in the laboratory samples, it had the highest value of all samples by nearly an order of magnitude in the field samples. All MC-LR levels were significantly different from each other (ANOVA, *p* < 0.05). The potential human exposure to MC-LR via the food chain was evaluated for the field samples (Table 1), and the highest exposure rate of 0.10 µg/kg of body weight/day was recorded for *O. sativa* BG358. 

## 3. Discussion

*M. aeruginosa,* the dominant bloom-forming cyanobacterium in fresh water environments in Sri Lanka, has the potential to produce the hepatotoxin MC-LR at levels as high as 340 µg/g on a dry weight basis under optimum conditions in culture [44,53]. Thus, the potential for exposure to MC-LR-contaminated water, plants, and fish through dietary sources is high [4,6]. Though several studies have reported human exposure to MC-LR via drinking water in Sri Lanka, nothing has been reported on the exposure via consumption of MC-LR-contaminated crops. Almost all types of crop plants require irrigation, and this could be a major route of MC-LR uptake by humans when agricultural land is irrigated with MC-LR-contaminated water [38,42]. During irrigation, some MC-LR may be degraded rapidly by exposure to sunlight or by bacteria in soil or water, but the half-life of MC-LR under natural conditions is about 10 weeks [54,55,56].

Though some MC variants have the ability to pass through the cell membrane via diffusion or penetration, the large molecular weight, 1000 Da, of MC-LR prevents it from crossing the cellular membrane to bioaccumulate [9,57]. Though potential MC-LR transporters, such as organic anion transporting polypeptides (OATPs), rat OATP1B2, human OATP1B1, human OATP1B3, and human OATP1A2, have been identified in animals, no MC-LR transporters have been found in plants [58,59,60]. However, the irrigation of plants with MCs-contaminated water has resulted in various cellular and tissue responses, such as reduced growth, reduced development, reduced photosynthetic activity, reduced productivity, increased oxidative stress and activations, changes in biotransformation systems, and death in plants. All of these responses are consistent with MC-LR accumulation within cells [61,62,63,64,65,66,67,68]. 

Most of the studies on MC-LR accumulation in crops have been short term; however, treatment with MC-LR-contaminated water during the whole growing season is a more realistic representation of natural conditions. Instead of using pure or crude extract of MC-LR, fresh bloom contaminated water was directly used in the laboratory setup to identify the potential of MC-LR accumulation in crops when available with other molecules and degrading microorganisms. *M. aeruginosa* cells were not lysed; we let the cells release the toxic material naturally. We were not able to detect protein-bound MC-LR, since plants and animals contain proteins phosphatases that can bind covalently to and sequester MC-LR [69]. Only free toxins found in plant tissues were quantified in the present investigation. Maintaining the same concentration of MC-LR over the entire four-month study duration under natural environmental conditions was difficult. Thus, the hydroponic systems received fresh, thick scum of *M. aeruginosa* daily, resulting in high MC-LR concentrations.

Each plant type in the laboratory study accumulated different MC-LR concentrations, although their exposure to MC-LR was identical. Similarly, environmental samples in the field study showed significantly different MC-LR accumulation levels despite being irrigated with similar MC-LR levels from the Padaviya reservoir. This is likely due to the plant species individual physiology, and *O. sativa* Suwandel, being a traditional wild type, may carry genes that are resistant to environmental or natural toxins. The metabolism of MC-LR within rice plants was observed [63]. This could explain the difference in the MC-LR concentrations in the two varieties of *O. sativa* that were exposed to the same MC-LR concentration in the present study. They may for example, reduce the MC-LR concentration via a glutathione-related pathway, which is one of the main detoxification processes in plants and animals [39,63,67,70]. 

All plants had a lower concentration of MC-LR in their tissues compared to their external concentration. The long distance transport of MC-LR to the edible parts of the plant could result in a partial breakdown of MC-LR. In addition, the inhibitory potential of MC-LR on protein phosphatases 1 and 2A and related enzymes could interrupt the bioaccumulation of MC-LR in rice grains and *I. aquatica* tissues. The photolysis and microbial degradation of MC-LR may considerably reduce the accumulation of lower concentrations of MC-LR in the plant tissues. 

The TDI of MC-LR recommended by the WHO is 0.04 µg/kg of body weight/day [9,10]. In the laboratory study, all the rice grain samples and the tissue samples of *I. aquatica* showed levels of MC-LR that significantly exceeded this TDI. In the case of BG358, the potential human exposure was >70 × higher than the TDI. If a person consumed any of these edible parts from this experiment, there would be a high risk of harm.

In the field study, the consumption of MC-LR-contaminated rice grains of BG358 could pose a health risk to humans. The potential exposure for humans consuming ~300g of this variety per day is 2.5 × higher than the TDI. Chronic exposure to MC-LR levels detected above TDI in the field study could allow MC-LR to concentrate in the body tissues over time, leading to considerable health risks [26]. While the MC-LR concentrations in Suwandel and *I. aquatica* harvested from the agricultural fields irrigated from Padaviya reservoir do not appear to pose human health risk if consumed at a normal rate, consumption of these plants above the average rate may be damaging. Furthermore, chronic exposure to lower levels may be a health hazard. Even at levels of MC-LR below the TDI, children may be at a relatively higher risk when they consume MC-LR-contaminated food [71,72]. MC-LR levels in the irrigation reservoirs of Sri Lanka are increasing [36,44]. Therefore, the possibility of considerable accumulation of MC-LR in rice grains and other irrigated crop varieties cannot be excluded in regions of Sri Lanka irrigated via freshwater reservoirs. 

*I. aquatica*, which is grown in the water along the reservoir bunds, is one of the main leafy vegetables consumed in the area. The field samples of *I. aquatica* were collected from the same location where people harvest, sell, and consume the plant. This situation further increases the health risk to the people in such areas, and they have a high potential to experience health issues induced by MC-LR, such as liver cancer, kidney damage, brain damage, and apoptosis in testes, due to long-term exposure both via drinking water and the food chain [4,16,17,18,19,20,21,22,23,24,25].

## 4. Materials and Methods

### 4.1. Chemicals and Reagents

MC-LR standards, HPLC grade methanol, acetonitrile (ACN), trifluoroacetic acid (TFA), 100% analytical grade methanol, and 70% methanol were purchased from Sigma-Aldrich, St. Louis, MO, USA. A Milli-Q water purification system was purchased from Millex; Millipore Corporation, Bedford, MA, USA.

### 4.2. Laboratory Study

Beira Lake (6°55′38″ N, 79°51′18″ E), Sri Lanka, is a hypereutrophic lake which is frequently covered by a thick cyanobacterial bloom. *M. aeruginosa* is the dominant cyanobacterium in the lake and contributes more than 95% of the total biomass of the phytoplankton community [43]. Fresh bloom material (cyanobacterial scum) was extracted from 10 cm below the surface at two sampling points, taking care to prevent cell lysis during collection, to prevent release of MC-LR into the water. The cyanobacteria-contaminated water was collected each day from June to October 2016, and it was applied to the plants at a rate of 0.33 L per plant.

Seeds of two rice varieties, a hybrid variety, BG358, and traditional rice variety, Suwandel, as well as *I. aquatica* and were purchased from Farmers’ Welfare Centre, Maharagama, Sri Lanka. Seeds were washed three times with tap water, washed two times with distilled water to remove matrix pollutants, and then hydrated for seven days in the dark on wet filter paper. After germination, seedlings were cultured in hydroponic tanks at 27 ± 2 °C, with a photosynthetic active radiation of 300 µmol photons m^–2^ s^–1^ and a photoperiod of 12 h. Thirty test plants were maintained in each hydroponic tank, and all positive controls received the Beira Lake water contaminated with fresh *Microcystis* material each day until the plant material was harvested. Negative control tanks received de-chlorinated tap water. The hydroponic tanks were maintained along with negative controls for each plant type under the same environmental conditions. All the plants were harvested at the end of their normal agricultural cycle, which was four months.

### 4.3. Field Study

Rice grain samples (variants BG358 and Suwandel) and *I. aquatica* plants were collected in autoclaved plastic bags from farmlands directly irrigated from the Padaviya reservoir in September 2016 in collaboration with the Farmers’ Welfare Centre, Padaviya. Sri Lanka. Water samples from the sampling sites for the Padaviya reservoir were collected from 10 cm below the surface into autoclaved glass bottles. Collected samples were transported to the laboratory and were stored at −20 °C until the MC-LR analysis. 

### 4.4. Extraction of MC-LR from Rice Grains

Rice shells were removed, and 20 g of rice grains were ground to a powder (n = 4) and mixed with 100% analytical grade methanol, followed by vortexing for 10 min (VELP Scientifica, Usmate, MB, Italy). Sonication (Elma, Singen, Germany) was conducted for 15 min, and samples were maintained in a shaker (Multi Shaker, EYELA, Tokyo, Japan) for 30 min to ensure the maximum extraction of MC-LR to methanol, and then the mixture was centrifuged (Biofuge Stratos; Heraeus, Hanau, Germany) at 12,000× *g* for 10 min. The supernatant was concentrated in a rotary evaporator (IKA, Labortechnik, Germany) at 40 ^o^C to remove excess methanol. The residue was reconstituted in 2 mL 100% HPLC-grade methanol followed by centrifugation at 13,000 ×*g* for 6 min [39]. The supernatant was filtered through a 0.22 µm Nylon filter (Millex; Millipore Corporation, Bedford, MA, USA) to remove solid material. The extraction procedure was repeated three times, and all extracts were combined.

### 4.5. Extraction of MC-LR from I. Aqutaica Plant Tissues

*I. aquatica* plants were rinsed thoroughly with distilled water three times and with 70% methanol to remove matrix contaminants. They were then dried on blotting paper. We collected 40 g samples (n = 4). MC-LR from plant tissue samples was extracted according to the procedure in Section 4.4 to conduct the HPLC quantification of MC-LR.

### 4.6. Extraction of MC-LR in Hydroponic System Medium and Water Samples from the Padaviya Reservoir

10 mL samples were used for the MC-LR analysis (n = 4). Samples were freeze-dried (ESCO, Changi, Singapore), and the residue was re-suspended in 2 mL of 100% HPLC-grade methanol and centrifuged at 13,000 × *g* for 6 min. The supernatant was filtered through a 0.22 µm Nylon filter to remove residues, and HPLC quantification was achieved for MC-LR using the prepared calibration curve [43].

### 4.7. Quantification of MC-LR 

The quantification of MC-LR in samples was performed using the Agilent 1260 HPLC instrumentation (Agilent Technologies, Palo Alto, CA, USA) according to a standard protocol [73]. The HPLC consisted of a pump, an auto sampler, a ZORBAX SB-C_18_ column (length 250 mm × 4.6 mm internal diameter × 5 μm particle size, serial number: USF0063623), and a photodiode-array (PDA) detector at 200–300 nm with a 3 nm resolution. The sample injection volume was 25 μL, and a gradient flow was maintained for the separation of MCs using a mobile phase; Milli-Q water—0.05% TFA, and acetonitrile—0.05% TFA. The temperature of the C_18_ column was maintained at 40 °C with a flow rate of 0.8 mL/min, and the system pressure was maintained at 1.5 atm [73]. The MC-LR concentrations of the prepared samples were determined using the standard curve. 

### 4.8. Statistical Analysis

Results were presented as the mean of four replicates with a control for each plant variety. All data are represented as mean ± standard deviation (SD). The significant differences of MC-LR concentrations were statistically evaluated by a two-way analysis of variance (ANOVA) by using MINITAB version 15 statistical software (MINITAB, State College, PA, USA), and differences were considered significant if *p* < 0.05.

### 4.9. Calculation

In Sri Lanka, the average daily hybrid rice consumption of a 60 kg adult is 300 g fresh weight [51]. The average daily per capita traditional rice consumption of a 60 kg adult in Sri Lanka is around 30 g [52]. The average daily consumption of *I. aquatica* by a 60 kg adult in South Asia ranges from 6 to 10 g [47]. The calculations were completed according to the following equation [39]:

(1) $$\mathrm{MC}-\mathrm{LR}\;\mathrm{intake}\;(µg/\mathrm{day}/\mathrm{person})\;=\;\frac{\begin{array}{c} \mathrm{Concentration}\;\mathrm{of}\;\mathrm{MC}-\mathrm{LR}\;\mathrm{in}\;\\ \mathrm{edible}\;\mathrm{tissues}\;(µg/\mathrm{kg}) \end{array}}{1000}\;\times \;\mathrm{Average}\;\mathrm{consumption}\;(g/\mathrm{day}/\mathrm{person})$$

Human health exposure via the consumption of MC-LR-contaminated rice and *I. aquatica* tissues could be determined as the amount of MC-LR in µg per one kilogram of body weight and in a 60 kg adult per day. The calculations were completed according to the following equation [39]:

(2) Human health risk (µg/kg)= MC−LR intake (µg/day/person)÷ Body weight of an adult (60kg)

## 5. Conclusions

The two variants of *O. sativa*, BG358 and Suwandel, and the leafy vegetable, *I. aquatica*, are among the main food sources in the Sri Lankan diet. The highest toxin accumulation was found in *O. sativa*, BG358 in the laboratory study. However, the highest MC-LR contamination from the field study was in *I. aquatica*, and the human exposure estimate remained just underneath the recommended TDI level, thereby not posing risks to human health unless it is consumed at higher rate than normal levels. BG358 poses the greatest health risk to humans because of the higher rate of consumption, while Suwandel poses the lowest risk, with MC-LR concentrations below the TDI. The human health risk via the consumption of these foods irrigated or grown in MC-LR-contaminated water may contribute to some of the health issues in Sri Lanka such as liver cancer and kidney injuries. Accordingly, irrigation water quality should be maintained closer to the potable water quality standards for MCs and other cyanotoxins in Sri Lanka. Therefore, a comprehensive evaluation of the presence of cyanotoxins in irrigation, drinking, and recreational water bodies in Sri Lanka is essential to reduce the hazards to human health in the future.

## Figures and Tables

**Table 1:** Mean concentrations of MC-LR and associated mean potential human exposure of *Oryza sativa* (variant BG358 and Suwandel) grains and tissues of *Ipomoea aquatica* in the laboratory study and field studies. Values shown are mean ± standard deviation (SD).

Experimental Design		Plant	Mean Concentration of MC-LR in Edible Tissues (μg/kg)	Mean Potential Human Exposure (µg/kg of Body weight/day)
Laboratory study	Positive control	*O. sativa* (BG358)	567.52 ± 4.88	2.84 ± 0.01
		*O. sativa* (Suwandel)	429.83 ± 4.39	0.22 ± 0.01
		*I. aquatica*	350.82 ± 2.86	0.06 ± 0.01
	Negative control *	-	ND	ND
Field Study	-	*O. sativa* (BG358)	20.97 ± 0.31	0.10 ± 0.01
		*O. sativa* (Suwandel)	18.19 ± 0.16	0.009 ± 0.005
		*I. aquatica*	132.86 ± 0.26	0.03 ± 0.01

ND: Not Detected. * Pooled data for all three plant types. Negative control: De-chlorinated tap water. Positive control for all plant types with fresh bloom of *M. aeruginosa* containing MC-LR (3,197.37 ± 1.04 µg/L).
